# Low-Bandgap Ferroelectric *h*-LuMnO_3_ Thin Films for Photovoltaic Applications

**DOI:** 10.3390/ma18051058

**Published:** 2025-02-27

**Authors:** Abderrazzak Ait Bassou, Lisete Fernandes, Denis O. Alikin, Mafalda S. Moreira, Bogdan Postolnyi, Rui Vilarinho, José Ramiro Fernandes, Fábio Gabriel Figueiras, Pedro B. Tavares

**Affiliations:** 1 Centro de Química-Vila Real, CQ-VR, Physics Department, Escola de Ciências e Tecnologia (ECT), University of Trás-os-Montes e Alto Douro, 5001-801 Vila Real, Portugal; jraf@utad.pt; 2 Centro de Química-Vila Real, CQ-VR, Unidade de Microscopia Eletrónica-Centro de Investigação e Desenvolvimento UME/CIDE, University of Trás-os-Montes e Alto Douro, 5000-801 Vila Real, Portugal; lisfernandes@utad.pt; 3 CICECO Aveiro Institute of Materials, Physics Department, Campus de Santiago, University of Aveiro, 3810-193 Aveiro, Portugal; denis.alikin@ua.pt; 4 Instituto de Física de Materiais Avançados, Nanotecnologia e Fotónica (IFIMUP), Departamento de Física e Astronomia, Faculdade Ciências da Universidade Porto, R. Campo Alegre, 687, 4169-007 Porto, Portugal; up201805011@up.pt (M.S.M.); b.postolnyi@fc.up.pt (B.P.); rvsilva@fc.up.pt (R.V.); ffigueiras@ua.pt (F.G.F.); 5 Centro de Química-Vila Real, CQ-VR, Chemistry Department, Escola de Ciências da Vida e do Ambiente (ECVA), University of Trás-os-Montes e Alto Douro, 5001-801 Vila Real, Portugal; ptavares@utad.pt

**Keywords:** low band gap, photo-response, ferroelectric materials, light harvesting, phot-active materials

## Abstract

This work explores the deposition of hexagonal (*h*-) LuMnO_3_ thin films in the *P6_3_cm* phase and investigates the conditions under which the synergy of ferroelectric and photoactive properties, can be achieved to confirm the potential of this material for applications in the development of next-generation photovoltaic devices. Single-phase *h*-LuMnO_3_ was successfully deposited on different substrates, and the thermal stability of the material was confirmed by Micro-Raman spectroscopy analysis from 77 to 850 K, revealing the suitable ferro- to para-electric transition near 760 K. Optical measurements confirm the relatively narrow band gap at 1.5 eV, which corresponds to the *h*-LuMnO_3_ system. The presence of domain structures and the signature of hysteresis loops consistent with ferroelectric behaviour were confirmed by piezoresponse force microscopy. In addition, light-dependent photocurrent measurements revealed the photoactive sensitivity of the material.

## 1. Introduction

Photovoltaics is one of the most highly regarded technologies for the future of the energy sector. Conventional photovoltaic systems based on semiconductors are limited in terms of efficiency and performance due to the bandgap of the materials [1]. Even though considerable progress is being made with halide-based materials some reliability challenges still need to be solved [2]. The need for innovative approaches is therefore repeatedly emphasised. In this context, photo-ferroelectric materials are gaining increasing attention due to their unique light-matter interactions, which can open up new possibilities for the next generation of photovoltaic technologies [3]. These materials can enhance the bulk photovoltaic effect (BPVE), in which the electric field from spontaneous polarization in ferroelectric materials separates electron-hole pairs without relying on a *p-n* junction [4]. This photo-response pathway means that the output photovoltage can no longer be limited by the material’s bandgap and can be increased by orders of magnitude [5,6,7,8]. Nonetheless, there is a development of a photovoltaic device based on ferroelectric materials, namely coordinating the generation of photocarriers and photocurrent for efficient charge collection at the electrodes [3]. The most important strategies include the design of heterostructures to exploit the BPVE [9,10], strain modification by epitaxial growth of ferroelectric films on different substrates [10,11] and bandgap engineering [12,13] to extend the absorption range into the visible region. Among these promising materials are the hexagonal manganite oxides, *h*-*RE*MnO_3_ (*RE* = Lu or Y), which exhibit a remarkable combination of properties [14,15]. The phase is thermodynamically stable up to 1300 °C and can be easily prepared as bulk ceramics by conventional techniques such as solid-state, sol-gel or co-precipitation [16]. These compounds can also be prepared as thin films using different deposition techniques namely: Metal Organic Chemical Vapor Deposition (MOCVD) [17], Pulsed Laser Deposition (PLD) [10,18] or RF magnetron sputtering. The ferroelectric transition occurs at very high temperatures (*T_C_* > 750 K) and the non-centrosymmetric *P6_3_cm* lattice structure exhibits asymmetric covalent bonding interactions between Lu and O along the *c* axis, which favours the off-centred electric dipoles responsible for ferroelectricity [15]. Last but not least, the bandgap (*E_g_*) is between 1.1 and 1.5 eV and thus within the optimal values for the utilization of solar radiation [19]. Therefore, this compound is particularly interesting for potential photovoltaic applications due to the BPVE. Also convincing is the fact that the *h*-LuMnO_3_ is one of the least studied compounds of the manganite family, especially the thin film phase grown on different substrates, and the comparison with monocrystalline or polycrystalline bulk systems [20]. The most commonly used substrates for the experimental deposition of thin films are the standard Pt/TiO_2_/SiO_2_/Si (100) substrates, as the Pt buffer offers high electrical conductivity and adequate thermal stability even in O_2_ environments. However, some problems can occur when the temperatures required for the growth of ferroelectric oxides are above 750 °C [21,22]. These issues may include diffusion of Ti and recrystallization of SiO_2_ affecting Pt microstructure and adhesion, leading to dewetting and porosity, a process that has been clearly proven with live imaging by confocal laser microscopy [23,24,25], ultimately hindering electrical characterization [17]. Solving this challenge is also essential for reliable applications of thin film oxides. An alternative substrate with Pt buffer is hexagonal sapphire, which has the advantage of only 0.9% lattice mismatch between Pt (111) and Al_2_O_3_ (001) [26]. This stability is extended to the high temperatures required to synthesize the functional oxide materials by various physical deposition techniques [5,10,26,27,28]. In this study, the synthesis of single-phase *h*-LuMnO_3_ thin films, deposited by the RF-magnetron sputtering technique on various substrates, including SiO_2_ glass, SrRuO_3_, and Pt(111)/Al_2_O_3_(001) is explored. Then, characterization will evaluate the quality of the film phase, particularly using Raman spectroscopy, for which there are few reports. Further measurements of electrical and optical properties include the evaluation of the electroactive response under illumination.

## 2. Experimental Procedures

A high-quality stochiometric LuMnO_3_ target with a diameter of 5 cm was prepared using auto-combustion sol-gel synthesis (urea), details can be found in [Supplementary Material]. The *h*-LuMnO_3_ thin films were deposited using an RF-magnetron sputtering system with a Vac Techniche RFG300VT (Saint Leonards/UK) RF power supply at 13.56 MHz. The Al_2_O_3_(001) substrates (Kristallhandel Kelpin) (Leimen, Germany) were cleaved into 10 × 10 mm^2^ pieces. The wafers were placed on the top of the resistive heater with a fixed distance of 8 cm between the substrate and the target. The temperature of the heater was measured with a type K thermocouple placed directly behind the substrate using a Eurotherm 3216 (Worthing, UK). Before each deposition, the chamber was evacuated below 5 × 10^−6^ mbar using a turbomolecular pump. During deposition, the argon pressure was set to 3.2 × 10^−2^ mbar, and manually controlled via a precision leak valve and a gas flow meter (Omega FMA 1700A/1800A model FMA 1812A-ST-Ar) (Amstelveen, The Netherlands). The Pt buffer layer (bottom contact) was deposited from a Pt target (*99.99%*) at 25 W RF power under argon at 0.045 mbar, onto the Al_2_O_3_(001) substrates at 400 °C for 5 min (~30 nm) and 15 min (>90 nm thickness). Subsequently, the Al_2_O_3_/Pt films were annealed at 800 °C to ensure their stability under the conditions of growing *h*-LuMnO_3_. The *h*-LuMnO_3_ films were deposited for 60 min at 60 W RF power, resulting in a deposition rate of ~7.5 nm/min without heating, and then annealed for 12 h in a tubular furnace with 1 bar argon free flow.

X-ray diffraction (XRD) patterns were acquired using a Panalytical X’Pert Pro MPD (Almelo, The Netherlands) equipped with an X’Celerator detector and a secondary monochromator, in Bragg-Brentano geometry, with *λ*(Cu_Kα1,2_) = 1.5418 Å, 2*θ* step size of 0.017° at 100 s/step. High Resolution (HRXRD) “Phi scans” measurements were performed using *Rigaku SmartLab* diffractometer, operating with λ(CuKα1/α2), or monochromatic λ(CuKα1) = 1.540598 Å radiation. The diffractograms were analyzed using X’Pert Epitaxy (Version 4.1) and OriginPro software (version 2018).

Scanning Electron Microscopy (SEM) and Energy Dispersive Spectrometry (EDS) were performed using an FEI Quanta 400 with W filament and an *EDAX* system respectively. The EDS acquisition spectra were performed at 15 kV to minimize the signal from the substrate and semi-quantification was performed standardless with ZAF correction factors without considering the elements from the substrates.

The unpolarized micro-Raman spectra were recorded in a backscattering geometry using the 633 nm He-Ne laser line for excitation and a 100× objective lens of a Leica microscope. The scattered light was analyzed with a Renishaw inVia Qontor spectrometer (Barcelona, Spain). Measurements were performed at room temperature and from 70 to 850 K with the THMS600 Linkam Stage. The laser power was adjusted to prevent sample heating. The spectra analysis of phonon modes was performed using a best-fit method of a sum of damped oscillators with the *IgorPro* software (version 6.0.0.0).

The diffuse reflectance measurements were carried out with *Varian* spectrophotometer *CARY* 50 (Agilent, Santa Clara, CA, USA) in a range from 200 to 1000 nm, using the BaSO_4_ standard compound as a white background reference. The acquired spectrum was converted using the *Kubelka-Munk* function, where the magnitude *F(R_∞_)* is proportional to the absorption coefficient (*α*). The optical bandgap (*E_g_*) is calculated according to the relationship introduced by *Tauc* and described by *Davis* & *Mott*: (*α*.E)^1/*n*^ = (*h.υ* − *E_g_*), where *E* = *h.υ* is the photon energy and *E_g_* is the optical band gap. The power-law exponent, *n*, depends on the transition type: *n* = 1/2 for a direct E*_g_* and *n* = 2 for an indirect *E_g_.* The value of indirect or direct *E*_g_ is estimated by plotting (*α.h.υ*)^1/2^ or (*α*.*h.υ*)^2^, respectively, as a function of the photon energy and extrapolating it when *α* is zero [17,18,29].

Piezoresponse force microscopy (PFM) was performed in an Ar-filled glovebox to avoid the influence of the humidity and was done using a scanning probe microscope NT-MDT NTEGRA-Aura (Amsterdam, The Netherlands) equipped with an external lock-in amplifier Zurich instruments HF2LI); and custom-built signal pre-amplifier; commercial probes from Budget Sensors: ElectriMulti75-EG, with Pt coating tips of radius of 25 nm, resonance frequency ~75 kHz and spring constant k around 3–5 N/m. PFM scans and spectroscopy studies were done using dual AC tracking of the first contact resonance (DFRT) [30]. A 2 V AC voltage was applied to scanning probe microscopy for scanning. Switching spectroscopy PFM was measured through the electrode to attest ferroelectricity and avoid the effects of the charge injection. The images were edited with *Gwydion* software (Version 2.67). Electrical measurements under illumination were done at the scanning probe microscope NT-MDT NTEGRA-Aura using a built-in current preamplifier with logarithmic transfer function (Supplementary Material) and back-to-back diodes in parallel protecting the electrical scheme. The measurements were done in capacitor geometry by the application of the voltage pulses to the Pt electrodes deposited at the surface. *Budget Sensors*: *ElectriMulti75-EG* cantilever with Pt coating was used for the measurements. To achieve strong contact 1 μN force was applied to the probe. The measurements were performed in dark conditions with a switched-off AFM laser and feedback held by the capacitive sensor. A commercial *US H-103D* xenon lamp was used to illuminate the film surface with UV light. The measurements were pre-calibrated by performing I-V measurements at 1 nm lift-off from the surface to confirm no spurious contributions to the current.

Electrical measurements as a function of temperature from 273 to 620 K, were made in a 4-electrode linear configuration, using a Keithley 6487 Picoammeter/Voltage Source, and the temperature monitored with a K-type thermocouple connected to a TM920C temperature reader. Electrical resistivity was calculated from the I-V curves by multiplying the resistance by the cross-sectional area and dividing by the length between the electrodes.

## 3. Results and Discussion

### 3.1. Structural Characterization

**Figure 1:**
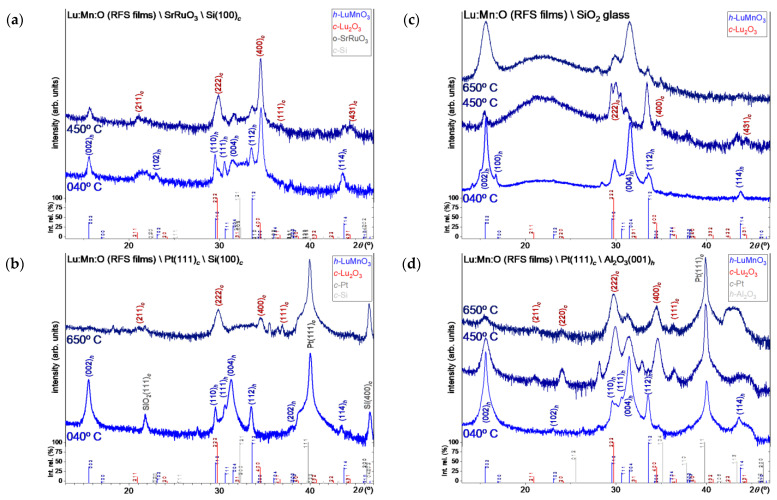
XRD patterns and indexations of LuMnO_3_ films deposited by RFS for 600 min at different temperatures followed by ex-situ annealing at 800 °C for 12 h in Ar. (**a**) on SrRuO_3_\Si (100)*_c_*; (**b**) on Pt(111)*_c_*\Si(100)*_c_*; (**c**) on SiO_2_ glass; (**d**) on Pt(111)*_c_*\Al_2_O_3_(001)*_h_*.

**Figure 2:**
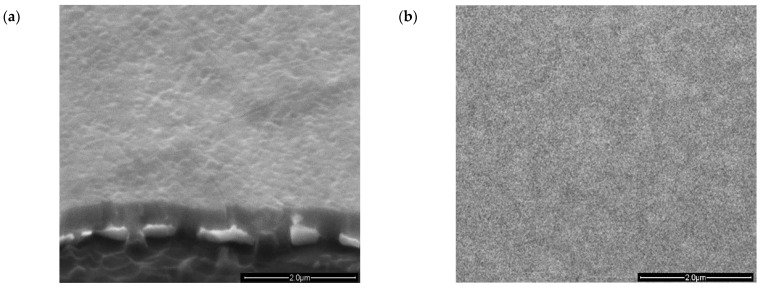
SEM images of the 450 nm thick *h*-LuMnO3 films deposited at 40 °C with ex-situ annealing at 800 °C for 12 h in Ar, (**a**) cross-section of film on Pt\Si substrate, and (**b**) surface of film on the Pt\Al_2_O_3_ substrate.

A series of Lu/Mn oxide films were deposited to determine the optimal conditions for the growth of the *h*-LuMnO_3_ phase. According to our previous work, *h*-LuMnO_3_ can only crystallize at temperatures around 800 °C [17]. Nevertheless, further experiments were carried out to investigate the influence of the substrate temperature during deposition on the development of the phase. Films were deposited for 60 min at 650 °C, 450 °C and at 40 °C, the inertial temperature of the plasma with the heater switched off. Then all were subjected to ex-situ annealing at 800 °C for 12 h in 1 bar Argon. Figure 1a–d shows the XRD patterns of the Lu:Mn:O films on different substrates. The diffractograms of samples deposited at 650 °C and 450 °C show mainly the presence of *c*-Lu_2_O_3_ (PDF#04-008-5231) [31] with clear reflection peaks at 2*θ* = 29.72° and 34.56 corresponding to planes (222)*_c_* and (400)*_c_* among other minor reflections from planes (211)*_c_*; (220)*_c_*; (111)*_c_*; (431)*_c_* confirming the formation of this phase. In addition, a mixture with an incipient phase of *h*-LuMnO_3_ can also be detected, with broad main reflections of the planes (002)*_h_* at 15.62° and (004)*_h_* near 31.45°. At such high deposition temperatures, the conditions for the formation of pure phase of *h*-LuMnO_3_ are not fulfilled. Consequently, it was found necessary to switch off the substrate heating and use only the inertial heating induced by the plasma (ambient conditions inside the chamber), which corresponds to stabilization at around 40 °C, monitored via a thermocouple probe in direct contact with the substrate. The film deposited on SrRuO_3_\Si (Figure 1a) still shows *c*-Lu_2_O_3_ as the dominant phase concomitant with the formation of polycrystalline *h*-LuMnO_3_. The film deposited on standard Pt\Si substrates (Figure 1b) appears more promising: It has no traces of *c*-Lu_2_O_3_ and a clear formation of the *h*-LuMnO_3_ phase with a preferential off-plane orientation along the *c_h_*-axis. However, the annealing treatment led to a slight segregation of the SiO_2_ from the substrate and a slight degradation of the quality of the Pt buffer, which affected the electrical characterization of the sample. A cross-sectional SEM image of this sample is shown in Figure 2a, from which the deposition rate of the film can be estimated. The film deposited on standard SiO_2_ glass substrates (Figure 1c) also appears to develop as the main phase of the *h*-LuMnO_3_ with a preferential off-plane orientation along the *c_h_*-axis. A small reflection peak at 2*θ* = 29.2° indicates traces of *t*-MnO_2_ from the (110)*_t_* planes [32]. Only the manganite film deposited on Pt(111)*_c_*\Al_2_O_3_(001)*_h_* (Figure 1d) shows no significant traces of spurious phases and therefore deserves special attention. It also shows a preferential orientation of the *h*-LuMnO_3_ *P6_3_cm* phase along the *c_h_*-axis, and observes intense off-plane reflection peaks from the planes (002)*_h_* at 15.62° and from (004)*_h_* at 31,46°, with an estimated lattice parameter *c_h_* =11.343 ± 0.001 Å, corresponding to a strain of −0.16% of the *c_h_* bulk parameter. Relatively weak peaks can be observed at 2*θ* = 23.07°, 29.57°, 30.61°, 33.55°, 43.58°, which can be indexed to the planes (102)*_h_*; (110)*_h_*; (111)*_h_*; (112)*_h_*; (114)*_h_* respectively. This allows the calculation of *a_h_* = 6.056 ± 0.004 Å in the plane, which compensates for an expansion of almost +0.3% with respect to the bulk parameter *a_h_*. Therefore, the film cannot be considered fully relaxed, and the average cell volume (V = 360.274 Å^3^) is within +0.4% of the typical dimensions of the fully stoichiometric bulk structure [33]. The SEM image of the surface of this sample shows a very homogenous microstructure, as can be seen in Figure 2b.

**Figure 3:**
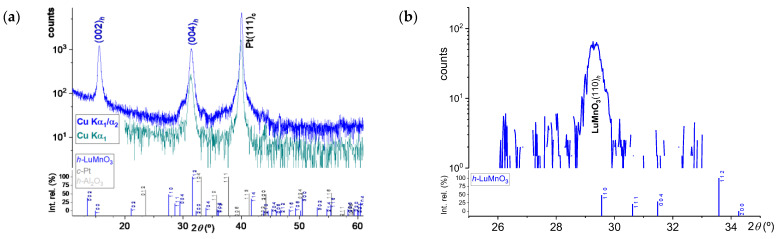
*θ*/2*θ* XRD scans of the *h*-LuMnO_3_ 75 nm thick film deposited at 40 °C on Pt\Al_2_O_3_ substrate, with ex-situ annealing at 800 °C for 12 h in Ar, (**a**) normal Cu Kα_1_/α_2_ and mono-chromated Cu Kα_1_ and (**b**) grazing incidence at *Phi* = 275° revealing the reflection of the in-plane {110}*_h_* planes.

As the previous series of *h*-LuMnO_3_ films were estimated to be about 450 nm thick, such broad films tend to relax the phase structure to approach the bulk form, masking possible strain effects resulting from the preferential crystalline orientation along the *c_h_*-axis on the Pt(111)*_c_*\Al_2_O_3_(001)*_h_* substrate. Further experimental depositions of thinner films were performed to determine whether the *h*-LuMnO_3_ phase can exhibit stronger more pronounced distortions due to epitaxial growth. Maintaining the previous deposition and annealing conditions, the new series of films was deposited for 10 min, corresponding to approximately 75 to 90 nm thin films, which are expected to be below or close to the structural relaxation threshold. XRD spectra shown in Figure 3a,b confirm a single-oriented growth of the film phase. For this thinner epitaxial film, the calculated lattice parameters a*_h_* = 6.090 ± 0.004 Å and c*_h_* = 11.384 ± 0.005 Å, which corresponds to a cell volume of 365.616 Å^3^, a relative increase of 1.92% compared to stoichiometric bulk structure [33]. The in-plane expansion of the cell parameters can be attributed to the epitaxial stress imposed by the sapphire substrate on the film, a similar effect that has been observed with other *h-RE*MnO_3_ compounds [34,35].

**Figure 4:**
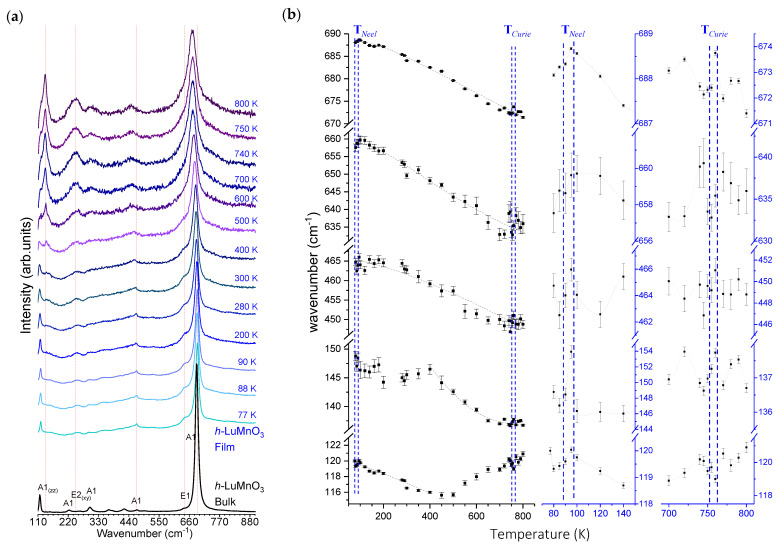
(**a**) Comparison of unpolarised Raman spectra of *h*-LuMnO_3_ bulk at room temperature [17,38] and of film with variation of temperature from 77 to 800K. (**b**) Respective evolution of reference Raman modes wavenumber with temperature. (**Insets**) Detail of the antiferro- to para-magnetic transition between 90–95 K and of the ferro- to para-electric transition between 755–765 K.

Further structural characterization was performed by unpolarized Raman spectroscopy. The *h*-LuMnO_3_ profile is known to have 38 Raman modes (9 A_1_ + 14 E_1_ + 15 E_2_), which are predicted by group theory for the irreducible representation of the Γ-point zone centre according to the equation [36]: Γ = 10 A_1_ + 5 A_2_ + 10 B_1_ + 5 B_2_ + 15 E_1_ + 15 E_2_. The details of 38 Raman active modes and their position for the *P6_3_mc h*-YMnO_3_ and *h*-LuMnO_3_ systems were studied by Iliev et al. [37]. Figure 4 a compares the spectra of the *h*-LuMnO_3_ film deposited in Pt\sapphire obtained in the range from 77 to 850 K with those of the bulk counterpart at ambient conditions [38]. Indeed, as detailed in Table 1, the film presents the expected coincident modes at 115, 461, 641 and 684 cm^−1^, while the main mode A_1_ mode at 684 cm^−1^ is slightly shifted to a lower value.

Figure 4b traces the most representative Raman modes and allows us to observe the evolution of the peak wavenumber and the bandwidths with temperature. The respective insets show the behaviour of these Raman modes between the temperatures of 90–100 K due to the antiferro- to para-magnetic phase transitions, and above 750 K starting the ferro-to para-electric phase transition. In particular, near 95 K, the 119, 466, 659 and 688 cm^−1^ modes exhibit a local maximum, indicative of the Neel temperature (T_N_), suggesting the presence of spin-phonon couplings in the AFM ordered phase. The anomalies of the E_1_ and A_1_ modes correlate with the displacements of Mn and O_2_ ions in the *c*-plane and Lu ions along the *c*-axis, in agreement with the literature [39]. As the temperature increases, there is a broadening of the Raman modes and some disappear, while others appear. Like the E_2_ mode near 135 cm^−1^, starting from 500 K, indicative of the involvement of added degree of motion of Lu(1) and Lu(2) atoms along the *c*-axis and the *ab*-plane respectively [37]. At temperatures above 755–760 K, significant changes are observed in the modes at 449, 634 and near 672 cm^−1^ going from A_1_ to A_1g_ and from E_1_ to E_1g_. But usually, in this range of high temperatures, the phase transitions occur gradually and are not sharply defined, hence the anomalies should be interpreted as a lower limit of Curie temperature (T*_C_*).

### 3.2. Band Gap Meausurements

**Figure 5:**
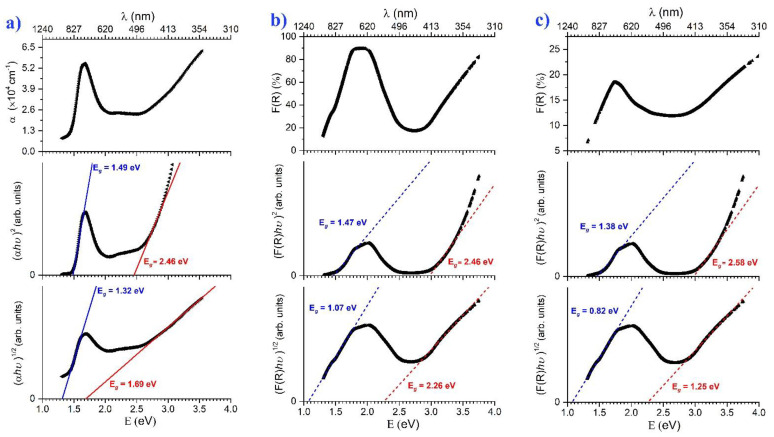
Measurements and calculation of direct and indirect optical Bandgap of the on SiO_2_ glass (**a**) by transmittance, and by diffuse reflectance on: (**b**) the ~450 nm *h*-LuMnO_3_ Pt\Al_2_O_3_; (**c**) The ~225 nm thick film on Pt\Al_2_O_3_.

Figure 5a–c shows the transmittance and reflectance measurements with the respective optical band gaps calculated using the *Tauc* method. The *h*-LuMnO_3_ films on SiO_2_ glass and Pt\Al_2_O_3_ substrates confirm similar results. The respective values estimated for the indirect gap are 1.32 and 1.07 eV within an error of 0.2 eV which might be related to the form of substrate used and the measurement techniques. While the direct gap is 1.49 and 1.47 eV respectively within an error < 0.005 eV. These narrow *E_g_* values are consistent with previous theoretical and experimental results [17,18,40,41,42]. The profiles of both curves’ show a strong absorption at low energies with a peak between 1.3 and 1.7 eV, followed by a slight decrease up to 2.3 eV, then a second strong absorption occurs. This behavior is a typical signature of *h*-LuMnO_3_ which is due to its electronic configuration and the resulting density of states (DOS) [17,18].

DFT calculations for *h*-LuMnO_3_ show that the valence band (VB) consists of the Lu *4f*, Mn *3d* and O *2p* states, while the conduction band (CB) is formed of the Mn *3d*, O *2p* and Lu *5d* states. Since the CB minimum and VB maximum are primarily dominated by the common Mn *3d* and O *2p* states [41,43], the inter-band optical transition from the occupied hybridized states of O *2p* with orbitals *d_xy_*/*d_x_^2^*_−*y*_*^2^* to the unoccupied Mn *d*_3*z*_*^2^*_−*r*_*^2^* states, requires relatively low energies (from 1.3 to 1.7 eV) for electrons to be excited into these available states [18,40]. Therefore, the first sharp increase in absorption. The CB DOS between 1.7 and 2.3 eV offers no available states for electrons, which means that photons in this energy interval cannot promote transitions and thus bypass the material leading to a decrease in absorption. At higher energies (>2.3 eV) absorption resumes as more available states of the CB become accessible for electron excitation. These are also attributed to an inter-band optical transition from the occupied hybridized states of O *2p* with orbitals *d_xy_*/*d_zx_* to the unoccupied Mn *d*_3*z*_*^2^*_−*r*_*^2^* states [40]. These secondary absorption thresholds on SiO_2_ glass and Pt\Al_2_O_3_ substrates indicate additional indirect band gap values of 1.69 and 2.26 eV, respectively and other direct band gaps of 2.46 and 2.98 eV, respectively, within a <0.01 eV margin.

These results show that the obtained band gaps are within the optimal absorption range of sunlight (1.1 and 1.4 eV) for photovoltaic applications [3]. The direct *E*_g_ transition dominates photovoltaic response due to its stronger light absorption and efficient charge carrier generation. While the indirect *E*_g_ can contribute at longer wavelengths, its phonon-assisted nature makes it less effective for PV performance. The direct transition enables a higher absorption coefficient. Thus, direct bandgap is the primary driver of photovoltaic activity, making *h*-LuMnO_3_ a promising material for solar energy conversion.

### 3.3. Piezoresponse Force Microscopy

**Figure 6:**
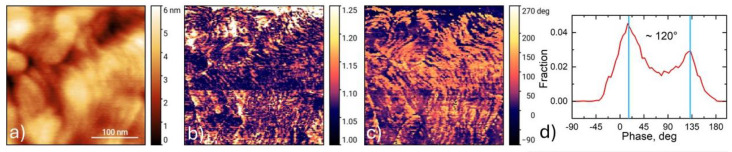
PFM scans for the 450 nm *h*-LuMnO_3_ film measured through top Pt electrode layer. (**a**) local topography, (**b**) amplitude and (**c**) phase contrasting domains. (**d**) phase histogram.

PFM characterization was employed to confirm the ferroelectric properties of the *h*-LuMnO_3_ films. The surface topography revealed an ensemble of crystallites with a size distribution between 30 and 100 nm as can be seen in Figure 6a. Both within and across each of the individual grains, the piezoresponse amplitude and phase scans, performed under 2 V_ac_, as depicted in Figure 6b,c, allow the observation of quasiperiodic patterns, that exclude substantial crosstalk effects to topography. The analysis of the PFM image histogram in Figure 6d, evidence that the phase difference between opposing oriented domains has around 120° phase shift, which can be influenced by the small size of the domains.

**Figure 7:**
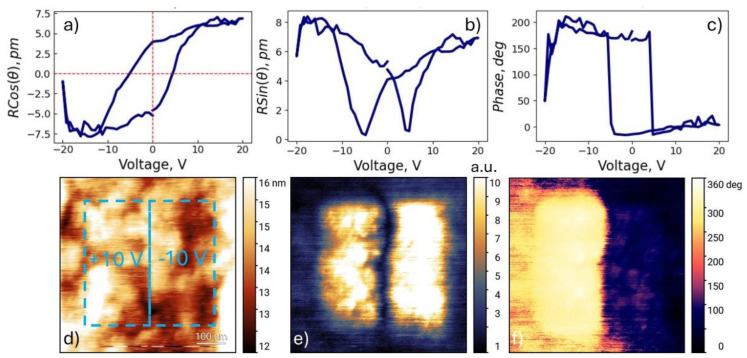
PFM ± 20 V_dc_ bias spectroscopy hysteresis loops of the 450 nm *h*-LuMnO_3_ film through top Pt electrode layer: (**a**) piezoresponse, (**b**) amplitude and (**c**) phase signal. Local bi-polar imprint pattern from scanning with SPM probe at ±10V_dc_ bias: (**d**) topography, (**e**) amplitude and (**f**) phase images.

Switching spectroscopy experiments were performed through Pt layer electrodes deposited on top of the film surface, in order to rule out effects related to charge injection artifacts. These measurements clearly observe polarization hysteresis loops with amplitude signal outlining a “butterfly”-like shape and the phase signal plotting a saturated “parallelogram”-shape as illustrated in Figure 7a–c. From these curves are estimated an effective piezoelectric coefficient around ~7 pm/V, and a coercive field near ~11 kV/mm. Local scanning with the probe biased at ±10 V_dc_ revealed clear imprint and polarization reversal, as observed in Figure 7d–f.

### 3.4. Electrical Characterization

**Figure 8:**
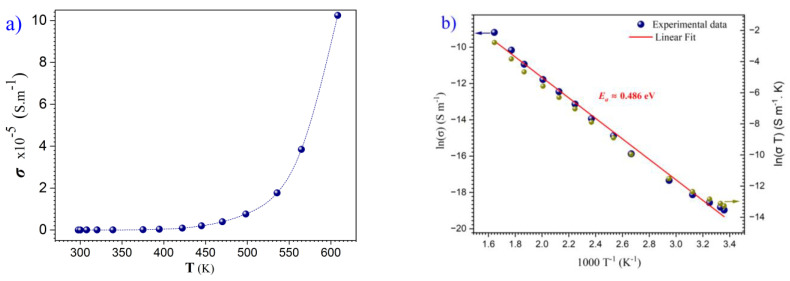
(**a**) Temperature dependence of the *h*-LuMnO_3_ thin film conductivity and respective (**b**) Arrhenius plot *ln*(***σ***) versus 1/T (Blue dots-left scale) and *ln* (σT) vs. 1/T (Green dots-right scale).

Since conventional measurements of the dielectric constant were inconclusive due to the high-loss behaviour of the samples, temperature dependence of the conductivity of the *h*-LuMnO_3_ film deposited on SiO_2_ glass can be observed in Figure 8a, indicating characteristics of a semiconductor-like behaviour, where the conductivity strongly depends on the availability of free charge carriers. In effect, the relatively low band gap < 1.5 eV means that at ambient temperature, a considerable number of electrons are thermally excited above the *Fermi*-level to reach the conduction band. The dependence of the conductivity (***σ***) on the temperature (T) follows the Arrhenius equation:

$$\sigma =A.\exp\left(-\frac{E_{a}}{k_{B}.T}\right)$$

where, *A* is a constant, *k_B_* the Boltzmann’s constant and *E_a_* the activation energy. This relationship can be seen in Figure 8b, where the plot of *ln*(***σ***) versus 1/T exhibits a clear linear trend, estimated from the slope, *E_a_* to be approximately 0.49 eV. i. e the minimum energy barrier that charge carriers must overcome to contribute to electrical conduction. The close agreement between the experimental data and the linear fit supports the validity of the model and shows that the conduction process is determined by the thermal excitation of charge carriers. This value agrees well with the activation energy, which is for BiFeO_3_ [44], BiMnO_3_ [45] and YMnO_3_ in the temperature range from 330 to 500 K with a value of 0.36 eV [46]. The conduction mechanism in this case is attributed to the thermally activated hopping of small polarons between localized positions of Mn^3+^ and Mn^4+^ [46,47] following Arrhenius function. This result is confirmed by the linearity of the plot ln (σT) vs. 1/T (green dots), validating thermally activated polaron hopping as shown in Figure 8b. The statement is supported by the results of the other ReMnO_3_ compounds form [46].

**Figure 9:**
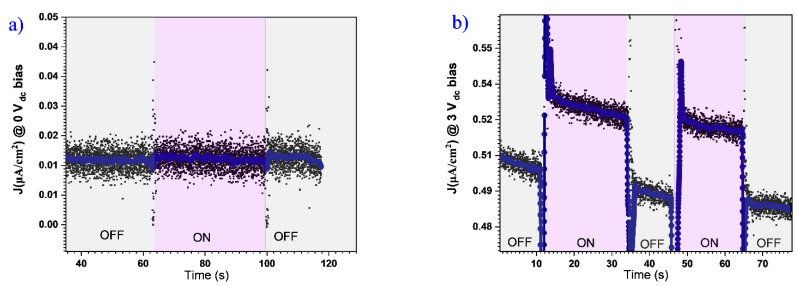
Photo-current measurements as a function of time through ON-OFF light states, with (**a**) no voltage and (**b**) 3 V bias applied to the system.

The photocurrent was measured both in the dark and under UV light at different applied voltages in capacitor geometry at the symmetric Pt/*h*-LMO/Pt dotted electrodes configuration. The graphs in Figure 9, show a residual current response ~0.02 µA/cm^2^, which is due to charge relaxation, when no voltage is applied between the electrodes. When a bias voltage of 3 V_DC_ is then applied, the sample shows a clear and consistent response to light, which is observed when it is successively switched on and off. The photocurrent in the ON-state increases by almost 0.04 µA/cm^2^ compared to the initial value in the OFF-state, indicating a clear photoactive response of the system. A slight decrease in current during the ON phase is likely due to depolarization which reduces the efficiency of charge separation. The peak oscillations between the ON-OFF events are associated with the acoustic noise generated by the mechanical shutter of the lamp and detected by the sensitive microscope. To rule out possible expansion of the sample due to heating from light exposure, the system was scanned continuously during ON-OFF switching cycles as shown in the line scans of Figure S5c, which confirms the stability of the topography under external illumination, as no amplitude or directional drift is observed.

This result is in close agreement with the results of similar materials, which emphasize the role of electric fields in enhancing light-induced charge separation and carrier transport. For instance, research on Bi_4_Ti_3_O_12_/TiO_2_ bilayers, for example, has shown that they exhibit an improved photocurrent response compared to single-phase films. Time-dependent measurements under different applied bias voltages showed that this effect can be significantly modulated by adjusting the ferroelectric polarization and external fields, which enables the effective separation of photogenerated charge carriers [48]. In addition, studies on thin films of Methylammonium lead iodide (MAPbI_3_) have shown transient photocurrent responses under bias voltage, which provide insights into the effective charge carrier dynamics [49]. In addition, studies on BiFeO_3_ films have shown that ferroelectric polarization and domain alignment under bias enhance the photocurrent response, highlighting the crucial role of ferroelectric properties in charge separation [50]. Thus, the *h*-LuMnO_3_ shows a similar behaviour, where the application of a bias voltage of 3 V probably aligns the ferroelectric domains, amplifying the intrinsic electric field and enabling efficient charge separation.

## 4. Conclusions

Single-phase photoactive *h*-LuMnO_3_ films were successfully deposited on platinized sapphire using RF magnetron sputtering, resulting in a high-quality homogeneous surface. XRD analysis confirmed the preferred *h*-LuMnO_3_(001) orientation for films below 90 nm thickness, while the structure gradually tends to other orientations for thicker films due to stress relaxation. The *h*-LuMnO_3_ phase films exhibited a narrow direct band gap below 1.5 eV, which is consistent with theoretically reported values [17,18,40,41,42]. Conferring a typical semiconductor behaviour with an activation energy of around 0.5 eV indicates that charge carriers are thermo-activated through the band gap and contribute to conductivity. Furthermore, PFM measurements also confirm the presence of a domain structure and ferroelectric hysteresis loops. Electrical measurements under illumination show consistent photoactive response, demonstrating the coupling of ferroelectric and potentially photovoltaic properties in a single material. The present results establish *h*-LuMnO_3_ as a promising candidate for advanced photovoltaic applications and pave the way for further research on its multifunctional properties and potential use in energy harvesting technologies.

## Figures and Tables

**Table 1:** Comparison of the Raman spectra modes central wavenumber for *h*-LuMnO_3_ at different temperatures.

Temperature (K)	A_1_	A_1_	E_1_	A_1_	Sample Type	Reference
90	119	482	--	690	bulk	[20]
	119	466	659	688	thin film	This work
300	117	463	640	689	bulk	[17,38]
	121	472	642	689	Single crystal	[36]
	118	469	648	688	thin film	[17]
	115	461	641	684	thin film	This work
750	--	--	--	682	Bulk	[20]
	--	449	634	672	thin film	This work

## Data Availability

The original contributions presented in this study are included in the article/Supplementary Material. Further inquiries can be directed to the corresponding author.

## Supplementary Materials

The following supporting information can be downloaded at: https://www.mdpi.com/article/10.3390/ma18051058/s1, Figure S1: Sintering procedure diagram used for the targets production. Figure S2: XRD pattern of LuMnO_3_ target with the reference *h*-LuMnO_3_ (Crystallography Open DB card n º. 9007909). Figure S3: (a) SEM image of the LuMnO_3_ target and (b) composition by EDS. Figure S4: (a) XRD patterns of Pt buffers deposited on Al_2_O_3_(001) for different deposition times; (b) *Phi*-scans of Pt film at 2*θ* = 46.32º and 67.58º. SEM images of the Pt films after annealing at 800° C for 12 h in 1 bar Ar (c) of 5 min deposition; (d) of 15 min deposition. Figure S5: I-V measurements performed under dark conditions, (a) symmetrical Pt/*h*-LMO/Pt configuration; (b) asymmetrical ITO/*h*-LMO/Pt configuration. (c) Stability of tip position during I-V measurements under dark and light conditions, revealing lines scanning without drift, excluding thermal dilation effects due to current or light heating.
