# Supplementary material for: Low-Bandgap Ferroelectric h-LuMnO3 Thin Films for Photovoltaic Applications
Source: Materials (Basel). 2025 Feb 27;18(5):1058. doi: 10.3390/ma18051058 (PMC11901307; doi:10.3390/ma18051058)
Supplement: Supplementary file 1 [file materials-18-01058-s001.zip › materials-3489708-supplementary.pdf]

Supplementary material

Low-bandgap ferroelectric *h*-$LuMnO_3$ thin films for photovoltaic applications

Abderrazzak Ait Bassou [1,*], Lisete Fernandes [2], Denis O. Alikin [3], Mafalda S. Moreira [4], Bogdan Postolnyi [4], Rui Vilarinho [4], José Ramiro Fernandes [1], Fábio Gabriel Figueiras [4] and Pedro B. Tavares [5]

Target preparation:

The 2-inch $LuMnO_3$ target was synthesised by the auto-combustion sol-gel method (*Urea*) from Mn (NO_3) $2.H_2O$ (*ABCR*) and Lu_2O_3 (*Alfa Aesar*) compounds. Each of the precursors was dissolved separately in deionized water adding the necessary HNO_3 solution at ~60º C in a goblet. The solutions are mixed and an amount of urea $(CO(NH_2)_2)$ whose mass is calculated as 3 times the mol number of the metallic ions (Lu + Mn), verifying full solubility and correcting the pH level to be around ~ 5.2 by slowly adding ammonia (NH_3) aqueous solution, while avoiding the formation of precipitates. The solution was then heated under stirring to evaporate all the water and wait for the urea decomposition, at the end of the process, when the temperature reached ~ 200ºC, the gel auto ignited and a fast controlled combustion (3 to 5 seconds) took place, resulting in a dark powder. This powder was moved to an alumina crucible and performed pre-calcination at 700º C for 2 hours in a furnace in the air. The calcinated powder was ground with a mortar and pestle and then sieved through a 38 µm mesh sieve; For target processing, moulding to 60 mm targets, axial pressing and sintering at 1400º C. The sintering process went through 6 annealing stages as presented in the plot of Figure S1. Starting with a 10 °C/min heating ramp to 700º C to minimize temperature gradients. After that, another heating ramp of 5 °C/min up to 1400º C, then kept for 48 h at 1400º C to promote grain growth and hardening of the target. Then, slow cooling down to 600º C for 12 h hours and kept there for 1 h to recover oxygen incorporation. Finally, proceed to slow cooling down during 7 h to room temperature. The quality of the target was characterized through XRD (figure S2), SEM (figure S3 a) and composition measured through EDS (figure S3 b).

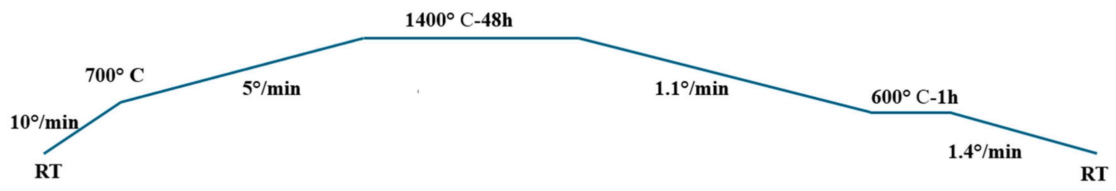

Figure S1: Sintering procedure diagram used for the targets production

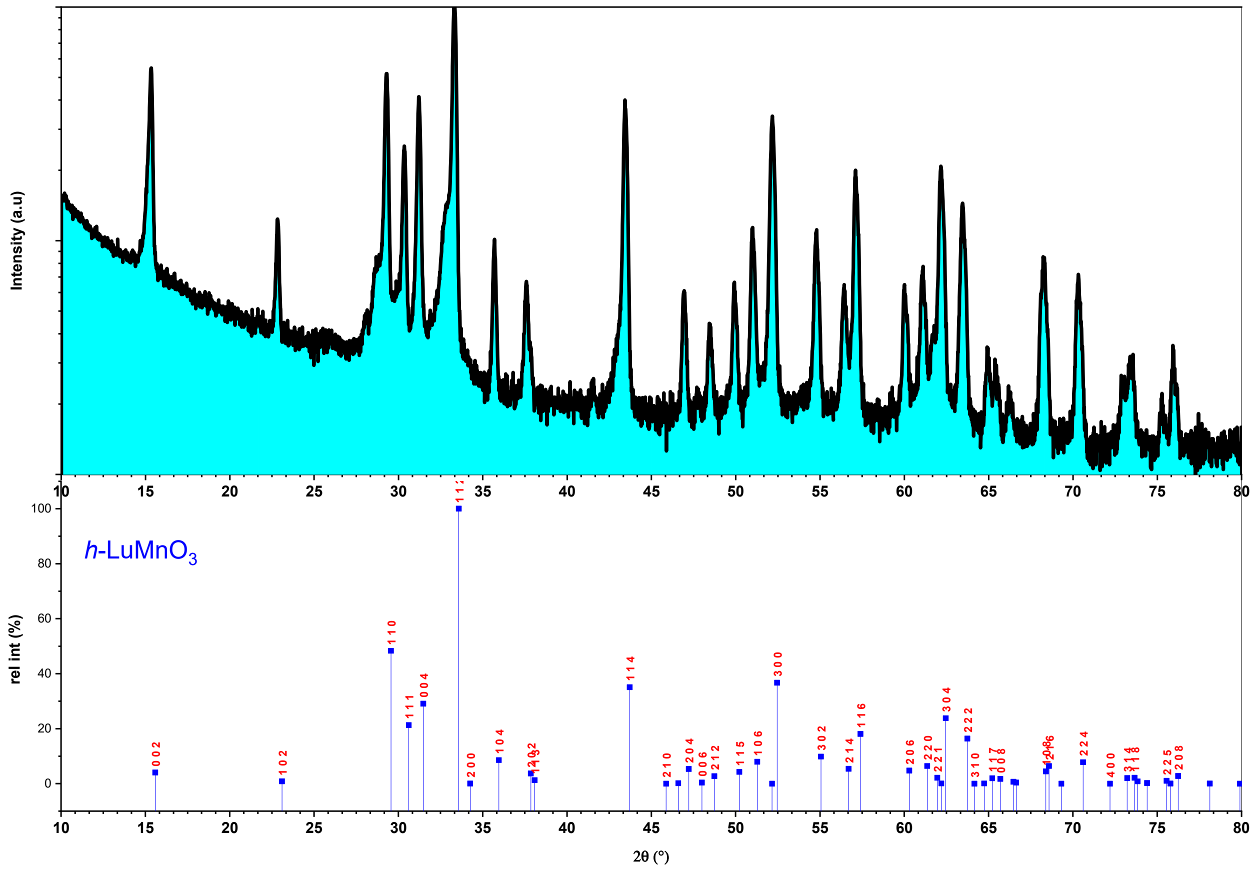

Figure S2: XRD pattern of $LuMnO_3$ target with the reference h-$LuMnO_3$ (Crystallography Open DB card n º. 9007909).

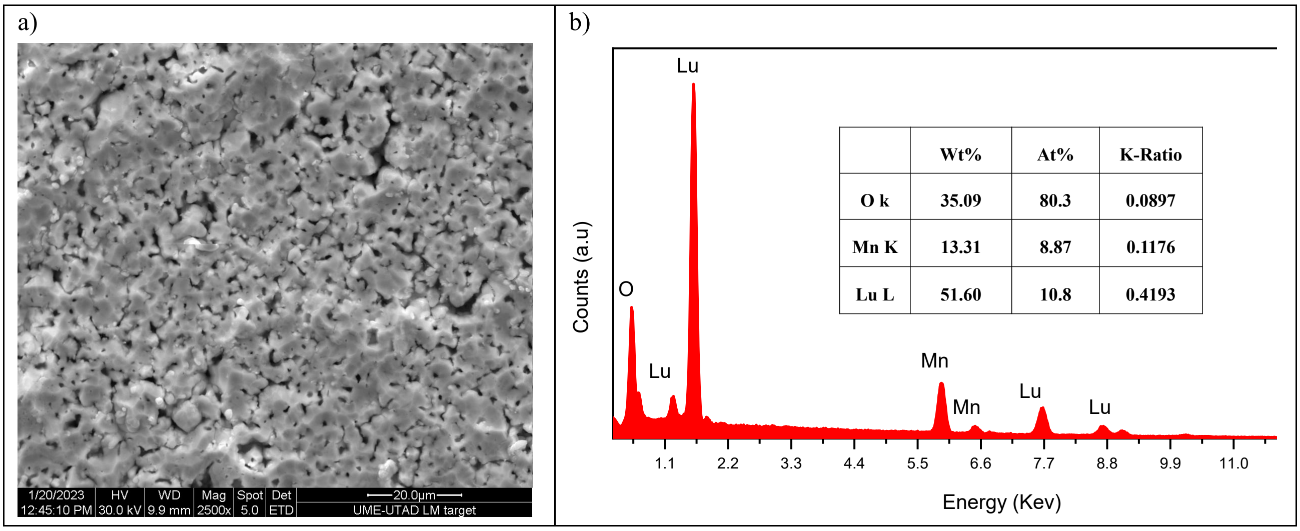

	Wt%	At%	K-Ratio
O k	35.09	80.3	0.0897
Mn K	13.31	8.87	0.1176
Lu L	51.60	10.8	0.4193

Figure S3: a) SEM image of the $LuMnO_3$ target and b) composition by EDS.

PT buffers XRD Characterization:

The XRD patterns of the control Pt buffer films seen in Figure S4 a) confirm the crystallization of the Pt phase. Both the 30 nm and 90 nm films have the main reflections of $(111)_c$ at 39.92° and $(220)_c$ at 67.605°, besides the Al_2O_3 substrate reflection at 41.48° for $(006)_h$. Figure S4 b) shows the in-plane XRD *phi*-scan spectra of the Pt film at $2\theta = 46.32^o$ corresponding to the six-fold symmetry (60° apart) family of planes $\{200\}_c$ and at $2\theta = 67.58^o$ corresponding to the twelve symmetry (30° apart) family of planes $\{220\}_c$. Both features can only be attributed to the cubic structure. The thickness of the films has relevant effects on the microstructure. As depicted in Figure S4 c), the thinner (~30 nm) film reveals some de-wetting from the substrate surface [16,17,23], resulting in a significant porosity of the Pt film. This effect is driven by the available diffusion mechanisms of the Pt layer, which rises with increasing temperature and annealing time. Hence, an increase in the film thickness can prevent the development of the de-wetting process [15,24]. Following this approach, the thickness of the Pt buffer was increased above 90 nm and revealed a dense and highly homogeneous microstructure as seen in Figure S4 d).

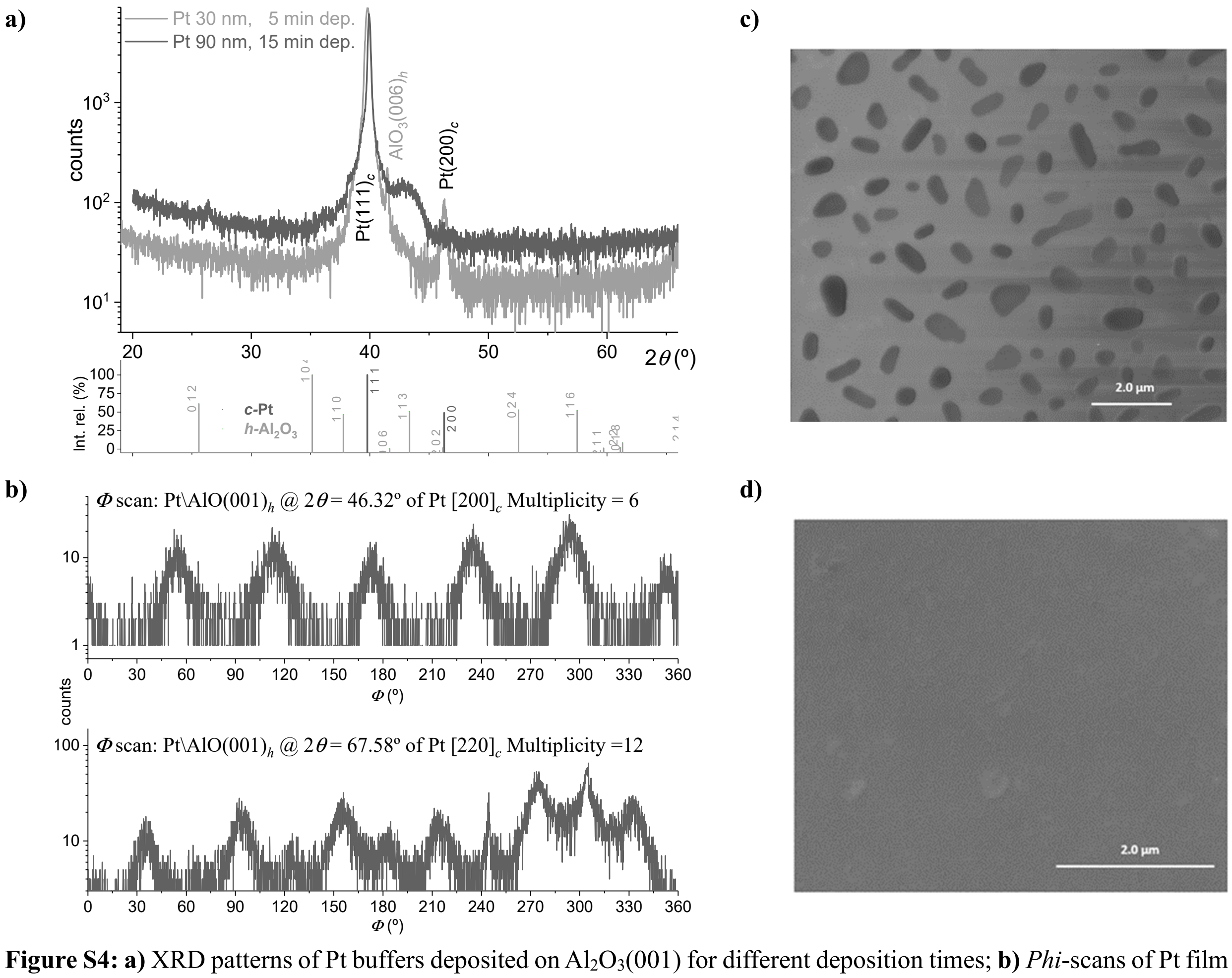

Figure S4: a) XRD patterns of Pt buffers deposited on $Al_2O_3(001)$ for different deposition times; **b)** *Phi*-scans of Pt film at $2\theta = 46.32^o$ and 67.58^o. SEM images of the Pt films after annealing at 800° C for 12 h in 1 bar Ar **c)** of 5 min deposition; **d)** of 15 min deposition.

Photo-electric activity measurements:

Two types of top electrodes were tested: a symmetrical Pt/*h*-LMO/Pt dotted configuration and a transparent indium tin oxide layer in an asymmetrical ITO/*h*-LMO/Pt arrangement. The first experimental step characterizes the electrical behaviour of the contacts by I-V measurements performed on the systems under dark conditions. Similar to findings reported in the literature [8], the results, shown in Figure S5, indicate that the top ITO/*h*-LMO contact exhibits an *Ohmic*-like behaviour (Figure S5 a), characterized by an electron-rich region. Conversely, at the bottom *h*-LMO/Pt electrode (Figure S5 b), the system demonstrates an electron-depleted region, forming a resistive *Schottky*-type contact.

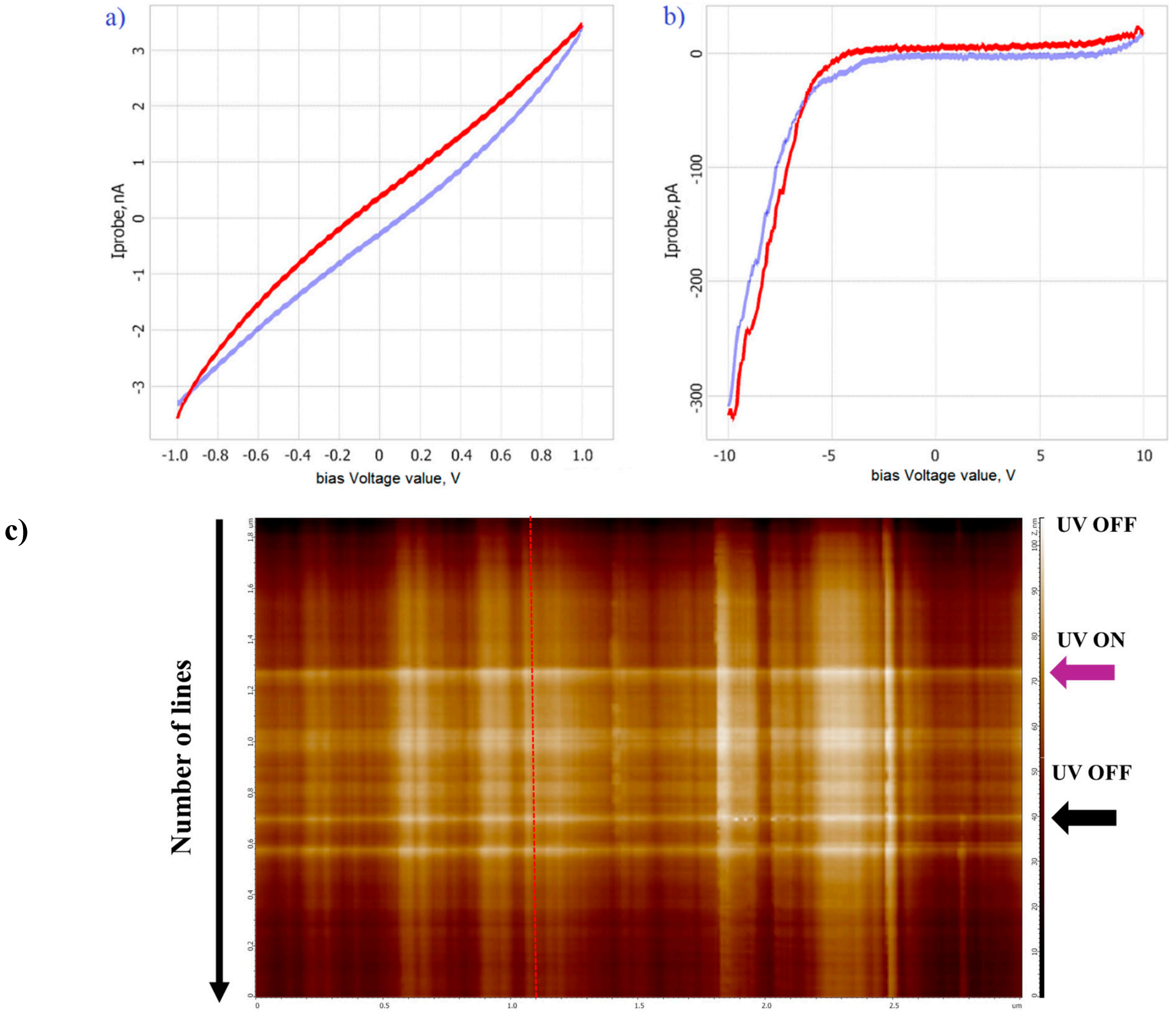

Figure S5: I-V measurements performed under dark conditions**, a)** symmetrical Pt/*h*-LMO/Pt configuration; **b)** asymmetrical ITO/*h*-LMO/Pt configuration. **c)** Stability of tip position during I-V measurements under dark and light conditions, revealing lines scanning without drift, excluding thermal dilation effects due to current or light heating.
